# The Effects of Safflower Yellow on Acute Exacerbation of Chronic Obstructive Pulmonary Disease: A Randomized, Controlled Clinical Trial

**DOI:** 10.1155/2019/5952742

**Published:** 2019-01-08

**Authors:** Xiao-jin Li, Yan Kang, Ru-rong Wang, Xue-lian Liao, Xiao-feng Ou, Jin Liu, Yun-xia Zuo

**Affiliations:** ^1^Intensive Care Unit, West China Hospital of Sichuan University, Chengdu 610041, China; ^2^Intensive Care Unit, The Third People's Hospital of Chengdu City, Sichuan 610031, China; ^3^Department of Respiratory Medicine, The People's Hospital of Pujiang County, Sichuan 611630, China; ^4^Department of Anaesthesiology, West China Hospital of Sichuan University, Chengdu 610041, China

## Abstract

**Objectives:**

To evaluate the efficacy of safflower yellow in the acute exacerbation of chronic obstructive pulmonary disease (AECOPD).

**Methods:**

In a prospective, randomized, controlled trial, 127 patients who met the inclusion criteria were enrolled and were randomly divided into two groups. The control group included 64 patients treated according to the global strategy for diagnosis, management, and prevention of COPD (www.goldcopd.org, updated 2011). The intervention group included 63 patients who received intravenous infusions of safflower yellow (100 mg of safflower yellow dissolved in 250 ml 0.9% saline) once daily for 14 consecutive days in addition to standard diagnosis and treatment. The difference in the average length of the hospital stay between the two groups of patients was determined. The follow-up period was 28 days; the differences in symptoms, clinical indicators, and 28-day mortality in the two groups were compared. Statistical analysis was conducted using SPSS 22.0 software to determine whether there were statistically significant differences (P <0.05) between groups.

**Results:**

There were no statistically significant differences between the intervention group and the control group in changes in secondary indicators. There were no statistically significant differences in the 28-day mortality or in the survival curves of the two groups (P=0.496 and P=0.075, respectively). Safflower yellow treatment of AECOPD may relieve the patient's clinical symptoms, such as dyspnoea, shorten the average length of hospital stay (P=0.006, respectively), and decrease the duration of mechanical ventilation.

**Conclusion:**

Safflower yellow in the treatment of AECOPD has a degree of clinical value. This trial is registered under the identifier ChiCTR-IPR-17014176.

## 1. Introduction

Globally, chronic obstructive pulmonary disease (COPD) is one of the leading causes of morbidity and mortality due to chronic disease. The social and economic burden imposed by COPD is increasing in part due to the ageing population. Based on studies conducted at global obstructive pulmonary disease institutions, it is estimated that, by 2020, COPD will be the third leading cause of death in the world and the fifth largest source of global economic burden due to disease [1]. COPD is a common, preventable, and treatable disease. In addition to smoking, airway or alveolar abnormalities can be caused by the exposure of individuals to toxic particles or gases, resulting in persistent respiratory symptoms and airflow obstruction [2]. The criteria for the diagnosis of COPD include patients with progressively worsening dyspnoea, persistent dyspnoea, aggravation of dyspnoea after activity, or repeated chronic cough and expectoration production and/or a history of exposure to risk factors, and pulmonary function test results showing that after the use of bronchodilators patients still have forced expiratory volume in one second (FEV1)/forced vital capacity (FVC) <0.7, confirming the existence of persistent airflow limitation [2]. As a result of global common concern, a “Global Strategy for the Diagnosis, Management, and Prevention of COPD” was produced by the Global Initiative for Chronic Obstructive Lung Disease (GOLD); this document was published as a 2007 edition [3], a 2011 edition [4], and a 2017 update [2]. With the economic growth of developing countries, industrialization, urbanization, and environmental pollution have occurred simultaneously. The increase in the levels of toxic and harmful particles and gases in the environment has increased the incidence and mortality due to COPD in developing countries, but the related clinical epidemiological and medical information is very limited [5]. The management of acute exacerbation of COPD (AECOPD) includes both nondrug treatment and drug treatment. Nondrug treatment includes rehabilitation, oxygen therapy, ventilation support, and surgical treatment. Drug treatment includes the administration of bronchodilators, glucocorticoids, antibacterials, and phosphodiesterase-4 inhibitors. The effect of currently available treatment regimens can only move patients from the acute exacerbation stage into a stable stage and cannot achieve the purpose of complete cure.

Chinese herbal medicine has been used in China for thousands of years. Because of the unique therapeutic effect of traditional Chinese medicine, it has won recognition from the Nobel Prize Committee [6]. Safflower yellow is the most effective component extracted from the traditional Chinese medicine safflower and is a natural platelet-activating factor (PAF) inhibitor [7]. PAF is an endogenous phospholipid mediator. It has been shown that many cells and organs can be stimulated in vivo by receptor-specific antigens to produce and release PAF. PAF acts not only on platelets but also on leukocytes, endothelial cells, lymphocytes, macrophages, and other cells and participates in the pathophysiological process of allergic asthma, acute inflammation, and other diseases. As a PAF inhibitor, safflower yellow has anticoagulant, anti-inflammatory, vasodilator, and antioxidant effects [8–11]. The recent research literature has shown that PAF receptor is a specific adhesion point of bacteria on cells, especially common haemophilus influenzae (NTHi) and streptococcus pneumoniae in the respiratory tract, which are prone to adhesion to small airway epithelial cells, the reticular basement membrane, and the alveolar space of COPD patients [12]. Given the increased platelet-activating factor receptor expression, an increase in the expression of the PAF receptor in promoting specific respiratory pathogens of acute and chronic respiratory infections is crucial. Some studies have evaluated the PAF receptor and phosphorylcholine in chronic obstructive pulmonary disease in the natural course of respective function and have discussed the airway epithelium PAF receptor and the bacteria-choline phosphoryl interaction as selective anti-infection targets in the potential treatment of chronic obstructive pulmonary disease [13]. Additionally, safflower yellow is a PAF receptor specificity antagonist and increases the circulatory anti-inflammatory effect. As such, the PAF receptor was the potential target for the treatment of patients with AECOPD, so we selected safflower yellow as a therapy to treat AECOPD. In our previous clinic trial, we also attempted to treat patients with severe sepsis and septic shock using safflower yellow and achieved good clinical anti-infection and anti-inflammation effects [11]. Clinical data on the use of safflower yellow in the treatment of AECOPD are sparse; furthermore, the existing studies often have ambiguous research designs and are not strictly prospective randomized controlled trials [14, 15]. We hypothesize that safflower yellow can partially reverse the pathophysiological process of COPD due to the obvious upregulation of platelet-activating factor receptor in the lung tissue of COPD patients, and safflower yellow's pharmacological effects include inhibiting inflammatory responses and oxidative stress, relieving pulmonary hypertension, reducing hypoxic pulmonary artery remodelling, and reversing right ventricular hypertrophy and remodelling [16, 17]. Therefore, a prospective randomized controlled trial was conducted in AECOPD patients in an agricultural county in western China from 2013 to 2015. In this trial, safflower yellow was used for intervention and treatment to improve the symptoms and signs of AECOPD, and the clinical effect of safflower yellow in treating AECOPD was evaluated.

## 2. Materials and Methods

### 2.1. Ethical Review and Registration of a Completely Randomized Controlled Clinical Trial

This randomized, controlled trial was reviewed and approved by the Ethics Committee of the People's Hospital of Pujiang County in Chengdu, Sichuan Province, China. The review number was Pujiang county ethical review 2013-A-1. This clinical study was registered on the designated website of the World Health Organization http://www.chictr.org.cn/showproj.aspx?proj=13739 under the registration number ChiCTR-IPR-17014176.

### 2.2. Selection of Research Subjects

#### 2.2.1. General Information

We collected patients with AECOPD who were admitted to the People's Hospital of Pujiang County. Each patient was informed of the purpose and methods of the study in writing and gave written informed consent to participation in the study prior to enrolment.

#### 2.2.2. Inclusion Criteria

Patients must meet the AECOPD diagnostic criteria of the 2011 American College of Chest Physicians and European Respiratory Society guidelines [4]. (1) Patients older than 40 years of age. (2) History: Any patients with dyspnoea, chronic cough, or increased sputum and with a history of exposure to risk factors. (3) Pulmonary function tests: post-bronchodilator FEV1/FVC <70%, that is, the presence of persistent airflow limitation, can be diagnosed as COPD. Confirmed diagnosis of COPD is based on the GOLD guidelines (post-bronchodilator FEV1/FVC 1/ ratio ≤70% and a post-bronchodilator FEV1 between 30% and 80% predicted).

#### 2.2.3. Exclusion Criteria

(1) Women who are pregnant, have recently given birth, or are breastfeeding. (2) Patients with coagulation dysfunction. (3) Patients with asthma or other lung-related diseases, such as lung cancer or active tuberculosis. (4) Patients allergic to safflower yellow. (5) Patients who are enrolled in other studies. (6) Patients with severe liver and kidney disease. (7) Patients with mental illness.

#### 2.2.4. Discharge Standards

(1) Symptom remission: remission of dyspnoea, chronic cough, sputum volume, wheezing and chest tightness, and fatigue. (2) Remission of complications: improvement of mental fatigue, anxiety, and depression. (3) Pulse oxygen saturation upon oxygen inhalation ≥92%. (4) Family members signed off to terminate treatment and requested discharge.

Prior to enrolment in the study, all patients and their families were informed of the specific details of the study and the fact that patients would be randomly included in either the intervention group or the control group. The control group was treated based on the diagnosis and treatment plans and measures set forth by the 2011 AECOPD International Guidelines of the American College of Chest Physicians (ACCP) and the European Respiratory Society (ERS) [4]; the intervention group was additionally treated with intravenous infusion of safflower yellow (100 mg dissolved in 250 ml of 0.9% saline) once a day for 14 days. All research procedures were conducted after obtaining written informed consent from the family members.

### 2.3. Evaluation of AECOPD

#### 2.3.1. Assessment of Lung Function

The degree of airflow limitation was classified based on the severity of pulmonary function, and the pulmonary function of COPD patients with airflow limitation was divided into four levels (Table 1).

#### 2.3.2. Acute Exacerbation Risk Assessment

The risk of acute exacerbation was assessed based on acute exacerbation history and pulmonary function assessment. Acute exacerbation involving two or more episodes in the previous year or FEV1<50% predicted suggests an increased risk.

### 2.4. Treatment

Based on a sequence of random numbers generated by the Statistical Package for the Social Sciences (SPSS) version 22.0 (SPSS Science Inc., Chicago, IL, USA) conducted by the statistician, the random seed was 20130101. All patients were included in either the intervention group or the control group by respiratory clinicians. The random numbers were stored in opaque, sealed kraft envelopes, with one number in each envelope, and the envelopes were opened in strict accordance with the time sequence of patient enrolment in the study. Each patient received one random number, and each random number was used only once during the entire study. The sequence did not undergo any modifications or adjustments. When the patient signed the informed consent form, the patient was informed that he would not know whether he received safflower yellow, and even the statisticians did not know which group was the intervention group.

The control group was treated according to the recommendations of the 2011 American College of Chest Physicians and the European Society for Respiratory Diseases AECOPD International Initiative. According to these standards, the goal of AECOPD treatment is to reduce the current clinical manifestation of acute exacerbations and prevent the occurrence of acute exacerbations in the future. The 2011 GOLD guidelines indicate that stable stage COPD can be treated with nondrug treatment and drug treatment. Nondrug treatment includes rehabilitation, oxygen therapy, ventilation support, and surgical treatment. Drug treatment includes drugs, such as bronchodilators, glucocorticoids, antibacterials, and phosphodiesterase-4 inhibitors (Guideline) [4]. The intervention group received intravenous infusions of safflower yellow (100 mg of safflower yellow dissolved in 250 ml 0.9% saline) once daily for 14 consecutive days. The treatment protocols are shown in Figure 2.

The independent investigators recorded the changes in secondary outcomes, such as respiratory rate, arterial oxygen partial pressure (PaO_2_), arterial carbon dioxide partial pressure (PaCO_2_), APACHE II score, complete blood count (CBC), liver and kidney function, and blood coagulation time, in the two groups. The independent investigators also recorded differences in indicators, such as average length of hospital stay, time on mechanical ventilation, and average hospital cost, in the two groups. The patients were followed up for 28 days from recruitment by telephone. Mortality in the two groups was observed, and the 28-day survival rate curves were plotted. SPSS 22.0 software was used for statistical analysis to determine whether there were statistically significant differences (*P*<0.05) between the groups and to assess clinical significance.

### 2.5. Treatment of COPD Complications

Cardiovascular complications include ischaemic heart disease, heart failure, atrial fibrillation, anxiety and depression, metabolic syndrome, and other concomitant diseases; all of these were treated according to the GOLD recommendations. If right heart failure occurred, intravenous injection of deacetylase lanatoside 0.2 mg + 20 ml of 0.9% saline was administered once daily until remission. If supraventricular tachycardia occurred, 10 mg diltiazem was administered orally three or four times a day or combined with 2.5 mg bisoprolol once daily to control ventricular rate. If anxiety occurred, 25 mg amitriptyline was administered orally once per night until remission.

### 2.6. Drug Sources, Drug Structure, and Scope of Use

**Figure 1:**
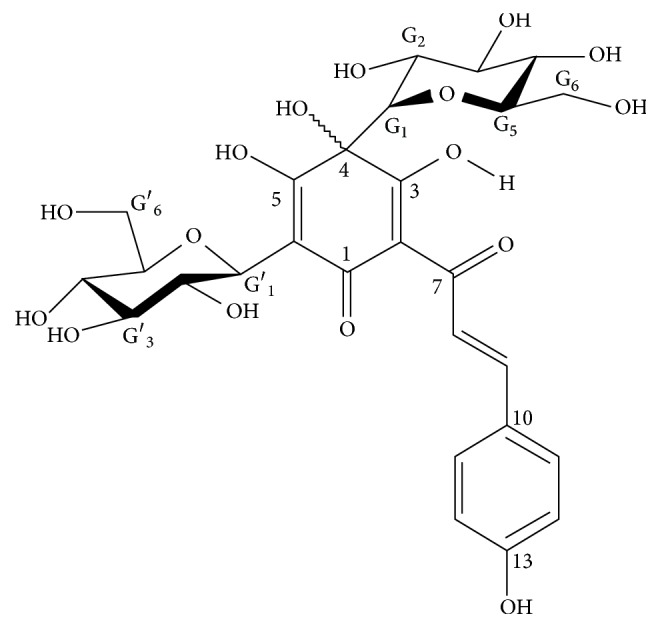
The molecular structure of hydroxysafflor yellow A.

**Figure 2:**
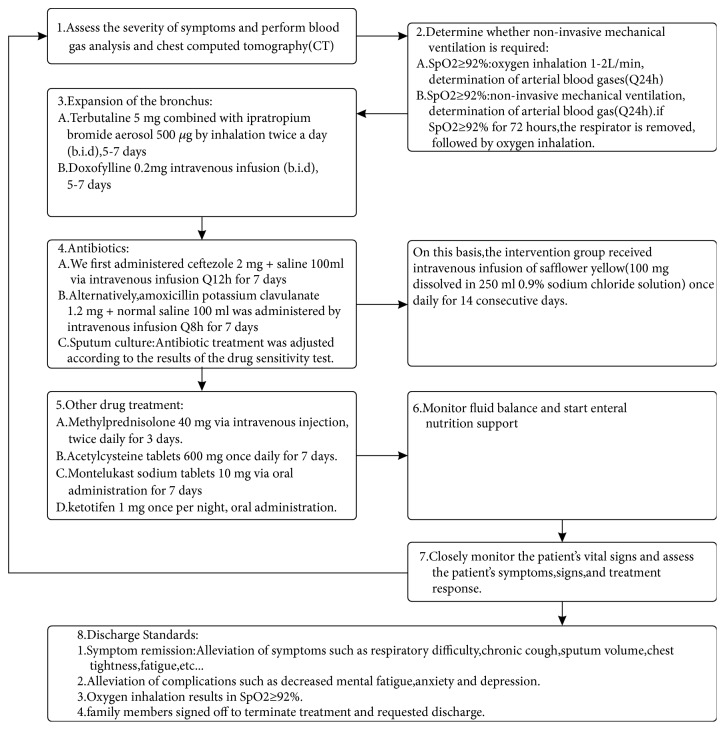
Therapy protocol for all patients.

The safflower yellow used in this study is an effective ingredient extracted from the traditional Chinese herbal medicine safflower. The safflower yellow was produced and manufactured by Zhejiang Yongning Pharmaceutical Co., Ltd., China. The Chinese medicine license number is Z20010146, and the patent number is ZL200810134819.1. The active group is hydroxysafflor yellow A [18]. The molecular structure of hydroxysafflor yellow A is shown in Figure 1. Its molecular formula is C_27_H_32_O_16_, and its molecular weight is 612.53 Daltons. Each 50 mg of safflower yellow contains 42.5 mg of the active ingredient hydroxysafflor yellow A. Hydroxysafflor yellow A has been used clinically in China for more than ten years and is mainly used for cerebral infarction and myocardial ischaemia. The active ingredient used in this study was 85 mg of hydroxysafflor yellow A administered daily.

### 2.7. Primary Outcome and Secondary Outcome

#### 2.7.1. Primary Outcome

The patients were followed up by telephone for 28 days. The average length of the hospital stay was taken as the primary outcome for both groups in our study.

#### 2.7.2. Secondary Outcome

Clinical symptoms, physical signs, and indicators before intervention, and after 72-hour, such as the mean arterial pressure, heart rate (HR), respiratory rate,* P*aO_2_,* P*aCO_2_, APACHE II score, CBC, liver and kidney function, coagulation time, and other intermediate indicators, as well as the mechanical ventilation time, average hospital cost, ventilator usage, cost-effectiveness analysis, 28-day mortality, charges per case, and 28-day Kaplan-Meier survival curve analysis, were used as secondary outcome.

### 2.8. Statistical Analysis

#### 2.8.1. Calculation of the Sample Size

This study was a completely randomized, double-blind (patients and statisticians were both blind to the trial design), controlled trial. Because the average hospital stay of previous AECOPD patients in our hospital was approximately 12 days, we used 12 days as the length of hospital stay for the control group. We assumed that the rate of loss to follow-up was 10%. After treatment with safflower yellow, the average hospital stay of the intervention group was expected to decrease by 30%, that is, to 8 days. The bilateral type I error *α* is 0.05, and the test effectiveness (1–ß) is 0.8. According to these four parameters, we calculated a necessary sample size of 140 patients (70 in each group) to detect a significant difference in the average hospital stay between the two groups.

#### 2.8.2. Statistical Analysis

Pearson's chi-square test or Fisher's exact probability method was used for count data. Normally distributed data were expressed as the mean ± standard deviation. One-way ANOVA or Student's t-test was used for measurement data. Non-normal data were compared using the Wilcoxon rank-sum test. The 28-day mortality test was conducted using log-rank Kaplan-Meier analysis of the logarithmic survival curve, and* P* values less than 0.05 indicated statistical significance. The statistical tool used was SPSS 22.0 (SPSS Science Inc., Chicago, IL, USA).

## 3. Results

**Figure 3:**
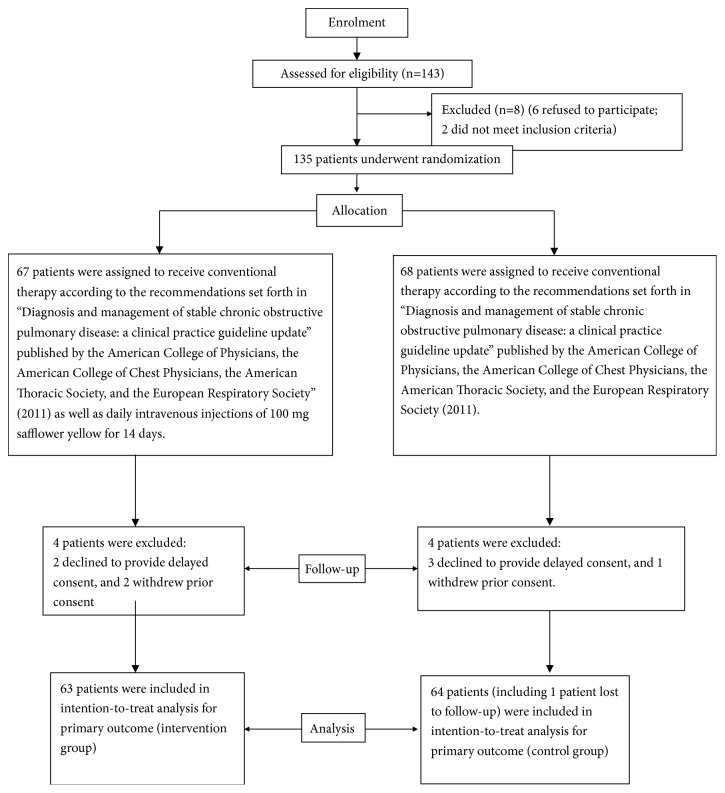
Patient flow diagram.

From March 1, 2013, to March 31, 2015, we collected and observed patients with AECOPD who were admitted to the Department of Respiratory Medicine of the People's Hospital of Pujiang County. During the 25-month study period, a total of 143 patients with AECOPD were admitted to our hospital. The study protocol is shown in the flowchart in Figure 3. Of these patients, 8 did not meet the inclusion criteria and were excluded, and 135 patients were randomly enrolled. According to the sequence of the random numbers, 68 of the 135 patients were included in the control group; these patients received routine diagnosis, assessment, and treatment according to the 2011 AECOPD International Guidelines of the ACCP and the ERS [4]. The goal of AECOPD treatment is reduction of the current clinical manifestations of acute exacerbations and prevention of the occurrence of subsequent acute exacerbations. A total of 67 patients were included in the intervention group, and all 135 patients were followed up for 28 days by medical professionals. In intention-to-treat analysis, there were 63 patients in the intervention group and 64 patients in the control group, including one patient in the control group who was lost to follow-up (Figure 3). There were 3 patients in the intervention group and 6 patients in the control group who voluntarily terminated all treatment due to personal reasons, such as heavy economic burden, poor financial status of the family, inability to pay for medical expenses, lack of willingness of the family members to support treatment, and low expectation of treatment efficacy. In the 127 patients, the family members of 3 patients in the intervention group signed and gave up treatment; this accounted for 4.76% of the sample size of the intervention group. In the control group, 6 patients gave up treatment; this accounted for 9.38% of the sample size of the control group. Two patients in the intervention group died within 28 days. To a certain extent, although some secondary indicators were clinically significant, they were not statistically significant. The patients who were lost to follow-up were treated as censored values, and the loss rate in the control group was 1.56%. The difference in voluntary abandonment rate between groups was not statistically significant (Table 3).

There were 69 patients with AECOPD alone; 36 of these were in the intervention group, and 33 were in the control group. Complications included cardiovascular disease, metabolic disease, anxiety, and depression, as shown in Table 1. We used computer-generated random numbers to randomly enrol patients in the intervention and control groups. The intervention group included 63 patients, and the control group included 64 patients. The two groups of patients showed no significant difference in terms of age, sex, weight, APACHE II score, FEV1/FVC after inhalation of bronchodilators, smoking rate, or incidence of complications. The two groups had the same baseline and were comparable (Table 2).

### 3.1. Main Outcome Findings

After the patients were enrolled and treated, the average length of stay of the two groups was compared; the mean hospital stay of the intervention group was significantly less than that of the control group (P=0.006; see Table 3).

### 3.2. Secondary Outcome Findings

**Figure 4:**
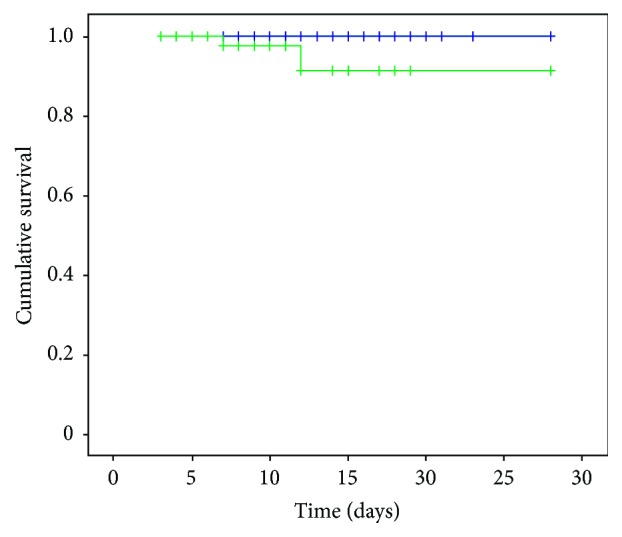
Kaplan-Meier survival curves of the intervention and control groups (P=0.075, all causes). Group 1: control group (blue line); Group 2: intervention group (green line).

Compared with the control group after treatment, the intervention group showed a decreased respiratory rate, increased arterial blood oxygen pressure, and decreased HR after treatment; the differences in these parameters were statistically significant (*P*<0.05). After treatment, the intervention group showed no significant decrease in platelet count, whereas the control group showed a decreased platelet count, with a statistically significant difference between the groups (*P*=0.002). In contrast, there were no statistically significant differences in intermediate indicators, such as the mean arterial pressure,* P*aCO_2_, white blood cell count or classification, liver or kidney function, or coagulation time (*P*>0.05) (Table 4). The average cost of hospitalization and the duration of mechanical ventilation were significantly lower in the intervention group than in the control group (*P*=0.001 and* P*=0.038, respectively) (Table 3). The cost-effectiveness analysis showed that the safflower yellow used in the intervention group cost RMB 65.5 per application, with a daily cost of RMB 131; thus, the actual treatment cost was RMB 1179 per 9 days. Considering that the intervention group saved RMB 2636 per patient, the cost-effectiveness ratio is (2636-1179)/1170 = 1.236, indicating a cost-effectiveness advantage. There was no statistically significant difference in the respirator usage rate, the 28-day mortality rate, or the 28-day Kaplan-Meier survival curve (*P*=0.693,* P*=0.496, and* P*=0.075, respectively) (Table 4 and Figure 4).

With respect to adverse reactions to the drug, one patient in the intervention group showed an allergic reaction to safflower yellow, with wildly spreading skin erythema, rash, and oedema. We immediately stopped the infusion of safflower yellow and intravenously injected calcium gluconate and dexamethasone. The allergic reaction was relieved, and the patient did not experience anaphylactic shock. There was no effect of the use of safflower yellow on liver function, as shown by the fact that no significant differences in liver function were observed after treatment (P=0.539) (Table 3).

## 4. Discussion

According to reports in the international literature, the occurrence of COPD is mainly related to smoking, burning of biomass fuel in densely populated areas, air pollution, and other risk factors [19, 20]. The smoking rates in the intervention and control groups of our enrolled COPD patients were 68.25% and 70.31%, respectively. Pujiang County is an agricultural county in western Sichuan province in China. Pujiang County belongs to Chengdu City and has a population of 260,500. The county's local fiscal revenue is 500.86 million RMB, and the per capita net income of farmers is 5094 RMB. Pujiang County is one of a number of impoverished counties in China. The number of beds in medical institutions in the county is 732. The number of beds per 1000 population is 2.8. Because Pujiang County is 80 kilometres from the provincial capital Chengdu, its residents' common illnesses are treated in the county hospitals. This gives our study a certain patient base, practical significance, and local characteristics. There is good ecological and environmental protection within the region; the PM2.5 concentration is 100 *μ*g/m^3^, and Pujiang County is one of the suburban counties of Chengdu City that has a relatively low environmental pollution index. According to statistics from our hospital, COPD is still one of the three most prevalent diseases among patients admitted to the Department of Respiratory Medicine in our hospital. In the UK, the British Thoracic Society has produced the second edition of “The Burden of Lung Disease” which reported the death of 27000 COPD patients each year [21]. Finding methods that can be used to prevent, assess, diagnose, and treat COPD, reduce the clinical manifestations of acute exacerbations, shorten the hospital stay of AECOPD patients, conserve limited national medical resources, and reduce the incidence of acute exacerbations has always been the goal of clinicians and of the Global Strategy for the Diagnosis, Management and Prevention of COPD [2]. In accordance with the international guidelines for clinical diagnosis and treatment requirements [4], we combined routine treatment with the use of safflower yellow and achieved a certain degree of clinical effect in this completely randomized, controlled clinical trial.

As we initially assumed, the results of this study generally reflected the therapeutic effects of safflower yellow by improving respiratory status, oxygen supply in arterial blood, and pulmonary hypertension and by increasing cardiovascular perfusion and cardiopulmonary function. This study showed that the use of safflower yellow significantly reduced the average hospital stay and the average hospitalization costs of patients with AECOPD, shortened the time for mechanical ventilation, reduced the per capita hospital expenses, and achieved a certain cost-effectiveness ratio, thereby reducing the economic burden on COPD patients.

Safflower yellow reduces cardiac ischaemia and increases heart function. Following the use of safflower yellow, AECOPD patients showed reduced myocardial ischaemia; safflower yellow also inhibited endothelin release, increased myocardial blood flow, improved myocardial oxygen consumption and metabolism, improved coronary perfusion, and reduced myocardial hypoxia, consistent with the results of previous studies [22]. In this study, safflower yellow decreased patients' HR and blood pressure. Safflower yellow blocks myocardial potassium channels, resulting in decreased calcium influx, which leads to reductions in left ventricular systolic pressure, left ventricular end-diastolic pressure, blood pressure, and HR [23]. Thus, safflower yellow maximally extends coronary diastolic perfusion time, dilates coronary arteries, increases coronary blood supply, and reduces left ventricular afterload. Safflower yellow can also inhibit hypoxia-induced apoptosis, increase nitric oxide content in hypoxia, and protect human umbilical vein endothelial cells from hypoxia by inhibiting apoptosis and cell cycle arrest. These findings may partially reveal the molecular mechanism of safflower yellow in the treatment of ischaemic heart disease [24]. In our study, we found that safflower yellow could dilate blood vessels, thereby lowering blood pressure, expanding coronary arteries, relieving myocardial ischaemia, and decreasing mental fatigue. This may be related to the fact that this drug can inhibit inflammation and oxidative stress, relieve pulmonary hypertension, reduce hypoxic pulmonary artery reconstruction, reverse right ventricular hypertrophy and remodelling, inhibit myocardial cell apoptosis, and improve cardiac ejection fraction [16, 17, 25], ultimately protecting and restoring cardiac function in patients with COPD and relieving heart palpitations and mental fatigue.

Safflower yellow increased PaO_2_, reduced respiratory rate, and improved hypoxia and dyspnoea in patients with AECOPD. Respiratory frequency was reduced after treatment with safflower yellow. The decrease in respiratory rate in the intervention group was significantly greater than that in the control group, and relief of dyspnoea was more pronounced in the intervention group. The decrease in the respiratory rate reduces the patient's WOB, relieves breathing difficulties, and reduces oxygen consumption by the heart and other vital organs. In addition, safflower yellow can reduce endotoxin-induced lung injury, increase PaO_2_, and decrease PaCO_2_. Compared with the control group, PaO_2_ increased significantly in patients in the intervention group. This may be related to the fact that safflower yellow can inhibit p38 MAPK channels, activate NF-*κ*B p65, and alter the expression of inflammatory cytokines [8]. Patients in the intervention group had increased oxygen supply to the organs, alleviating hypoxic symptoms and signs. This may be related to the ability of safflower yellow to alleviate hypoxia-induced pulmonary hypertension and reverse the effects of right ventricular hypertrophy [16]. Hydroxysafflor yellow A (CAS number is 78281-02-4) is a major ingredient in safflower yellow, and hydroxysafflor yellow A represents the most abundant and active components in safflower yellow. The main pharmacological effects of safflor yellow A are that it is an antagonist for platelet-activating factor receptor, and platelet-activating factor receptor in the lung tissue of patients with COPD was obviously upregulated [11], which increased airway inflammation, so we used hydroxysafflor yellow A to block upregulated platelet-activating factor receptor in AECOPD, which reduced pathogen adhesion and airway response. Furthermore, T lymphocyte apoptosis was inhibited by safflower yellow [26]. Safflower yellow also enhances the body immunity; some data also supported that inhibiting tyrosinase activity is achieved by anti-inflammation and antioxidation effects [27]. The combined effect alleviates symptoms in AECOPD patients, shortens the hospitalization and mechanical ventilation time, reduces the average hospitalization cost, decreases the burden on the National Health Insurance Fund of China, conserves medical resources, and improves prognosis.

The literature shows that platelets are cells that promote blood clotting as well as immune and inflammatory cells and play an important role in both innate and acquired immunity. In acute inflammatory and infectious diseases, increases in the number of platelets, the production of diverse platelet-derived inflammatory mediators, and multiple interactions between platelets and other cells directly or indirectly enhance the effects of platelets on immunity and inflammation [28]. In this study, we achieved a better understanding of the important effects of platelets in protecting and regulating immune function in COPD. In these patients, safflower yellow improved the platelet index. There was no significant reduction in platelet count in the intervention group, but the control group displayed high levels of platelet activation, greater platelet consumption, and significantly reduced platelet counts. The results suggest that safflower yellow inhibited platelet activation and aggregation in the intervention group, possibly by blocking the activation of the coagulation factor cascade in patients with COPD. Safflower yellow, therefore, decreases the negative effects of activated platelets in inflammation, enhances the immunoprotective function of platelets, reduces the inflammatory injury to blood vessels caused by platelet-secreted inflammatory molecules, such as platelet factor 4 (PF4) [29–31], protects vascular endothelial cells, promotes pulmonary blood circulation, improves oxygen supply and oxygen consumption, and reduces the patient's symptoms and signs, effectively shortening the average length of stay and reducing the amount of time for which mechanical ventilation is required.

In summary, the key finding of this study is that safflower yellow may improve pulmonary hypertension and right ventricular failure in AECOPD patients, thereby alleviating systemic hypoxia and, in particular, improving myocardial ischaemia and reducing WOB. Administration of safflower yellow also shortens the patients' average hospital stay, decreases the average hospitalization cost, reduces the time for which mechanical ventilation is required, achieves a certain level of cost-effectiveness, and has certain clinical significance.

The mortality rates and the 28-day survival rate curves of the two groups did not show statistically significant differences, probably because the AECOPD condition of the enrolled patients was mild to moderate; the average APACHE II score was 12 points. The respirator usage rate of both groups was approximately 20%. During the 28-day follow-up period, 2 patients in the intervention group died. This was not the result of the safflower yellow infusions. One reason for death was that the patients were critically ill, and family members of the 2 patients in the intervention group signed and gave up treatment. When the patients returned home, the patients died from respiratory failure, and no patients in the control group died; thus, the mortality rate was low. There are several possible reasons for this. First, some patients with severe COPD were admitted to the emergency department and treated with invasive ventilation therapy, such as endotracheal intubation. According to our observations, if the patient was affluent, family members typically request that the patient be transferred directly to a hospital with better medical facilities, such as the West China Hospital of Sichuan University, to receive further treatment, whereas the family members of poor patients often choose to abandon all treatment measures in the emergency department and go home. The patients who make up this subgroup of critically ill patients cannot be included in this study; this is one of the reasons that the AECOPD patients admitted to our hospital suffered from less severe disease. Second, most of the patients in this study live in poor rural areas and have a low level of education. Most of the patients have only graduated from primary and secondary schools, and some of them are illiterate. Little is known about the prevention of AECOPD, and patients are resistant to using basic emergency room medical equipment, such as noninvasive ventilators. Some patients also have a claustrophobic fear of wearing a mask and do not follow the doctor's advice well, resulting in poor compliance to treatment. Despite our best efforts to follow the protocol and to communicate and explain the procedure to the patients, the rate of ventilator use in the two groups of patients was only approximately 20%. When executing the guidelines, we still experience a certain degree of difficulty and a gap in implementation compared with foreign developed countries [32, 33]. Third, we attempted to implement the international guidelines by applying a variety of treatments, including termination of smoking, improving nutritional status, strengthening enteral nutrition support, controlling respiratory diseases, improving physical rehabilitation activities, especially activities designed to restore diaphragmatic function in patients with COPD, and conducting cardiopulmonary rehabilitation training activities, such as backslapping on sputum drainage, vibration sputum drainage, postural drainage, and acupuncture and moxibustion to promote the patient's early recovery and discharge. However, the medical conditions, medical care, and medical facilities in the Pujiang region have fallen behind international standards. We lack complete rehabilitation training equipment and advanced-treatment drugs, and advanced methods of treatment are not available [34–38]. Despite this, we used safflower yellow to treat patients with AECOPD and significantly shortened the hospital stay of AECOPD patients, reduced the average hospital cost, shortened the mechanical ventilation time, and achieved good cost-effectiveness. The use of safflower yellow in the treatment of AECOPD, thus, conserves limited medical resources and creates certain medical and social benefits.

In China, safflower yellow is widely used to treat ischaemic angina pectoris and acute cerebral infarction in coronary heart disease over one or two decades (the Chinese medicine license number is Z20010146), and it has been shown to be safe and effective in clinical use [39, 40]. In this study, safflower yellow did not affect the liver or kidney function of patients and did not cause organ damage. Although there was one case of safflower yellow allergy in the intervention group, it was found and treated promptly, and no serious complications occurred. We believe that safflower yellow is safe and effective when used in the clinical treatment of AECOPD.

The limitations of this study are as follows. (1) This study is a single-centre, completely randomized, controlled study that did not use the three-blind or the double-dummy technique. The physicians and patients who participated in the study were residents of a poverty-stricken agricultural county in western China with a population of 260,000. Severely ill patients were transferred to a higher-level hospital; thus, the representativeness of our clinical samples is limited. Compared with international multicentre, large-sample, three-blind trials, the gap between our study and other studies in scientific research methods and sample inclusion is obvious [41, 42]. As a next step, we plan to collaborate with large hospitals in a multicentre, large-sample, completely randomized, clinical controlled study to further evaluate the clinical value of safflower yellow in the treatment of AECOPD. (2) Our clinical studies were conducted in an agricultural county in a backward region of western China. The economic basis of this region is relatively low. Despite our attempts to follow the requirements of the international guidelines, there is still a gap between the medical technology and the inspection methods in use in our hospital and the guidelines prescribed by international initiatives. As a poor region in the western part of China, the clinical treatment effect, the scientific research management model, and the medical compliance of patients with COPD lag significantly behind those in developed countries [43, 44]. (3) In this study, patients and their relatives from rural areas lacked knowledge of methods for the prevention and medical assessment of COPD and had high expectations of the effects of clinical treatment. If there was no obvious relief of symptoms after a few days of treatment or if the treatment resulted in higher medical costs, family members often chose to abandon treatment. The rates of abandonment of treatment in the intervention group and the control group were 4.76% and 9.38%, respectively. The outcome of inadequate treatment may have some impact on the results. (4) Physicians in primary hospitals in China focus on alleviating the symptoms and signs in patients with AECOPD. They tend to focus on treatment measures and to pay little attention to prevention, assessment, rehabilitation, and follow-up work. The forms were completed without details and the specificity, sensitivity, and accuracy of the diagnoses were not studied in depth, and no analysis of whether the COPD cases involved complicated diseases, such as asthma-COPD overlap syndrome, was conducted; thus, this study lags behind similar studies abroad [45–47]. (5) This study observed the clinical effects of safflower yellow on the treatment of AECOPD. Due to the limiting conditions of our study, we did not collect samples for cytology or molecular biology research and did not attempt to identify the therapeutic targets or the molecular mechanisms through which safflower yellow is effective in the treatment of AECOPD. We believe that it is necessary to explore the molecular mechanism of action of safflower yellow in the treatment of AECOPD.

## 5. Conclusion

The use of safflower yellow in the treatment of patients with AECOPD significantly shortened the average hospital stay of patients, reduced the average cost of hospitalization, shortened the mechanical ventilation time, reduced treatment cost and the consumption of medical resources, and achieved a certain cost-effectiveness ratio. The research results obtained in this study provide a potential new strategy for treating AECOPD patients in poverty-stricken regions of developing countries more effectively. Based on these results, we plan to conduct a multicentre, large-sample, three-blind, double-dummy, completely randomized controlled clinical trial with increased sample size to explore the clinical effects of safflower yellow on the treatment of AECOPD. We will also utilize molecular biology techniques to conduct in-depth explorations of the molecular mechanisms, targets, and pathophysiological changes involved in drug therapy.

## Figures and Tables

**Table 1:** Classification of airflow limitation in patients with COPD.

FEV1/FVC <70% (FEV1 after inhalation of bronchodilators)	

GOLD1: Mild	FEV1 ≥80% predicted
GOLD2: Medium	50%⩽ FEV1% <80% predicted
GOLD3: Severe	30%⩽ FEV1% <50% predicted
GOLD4: Extremely severe	FEV1% <30% predicted

**Table 2:** Characteristics of the trial patients at baseline.

Demographics	Intervention group (*n* = 63)	Control group (*n* = 64)	*P* value
Age (years)	70.14±10.78	72.48±9.45	0.195
Weight (kg)	53.08±6.22	53.41±6.00	0.764
Sex (male:female)	37/26	35/29	0.721
FEV1 (%)	67.42±12.03	68.47±13.67	0.646
Smoker/non-smoker	43/20	45/19	0.801
APACHE II	12.06±2.56	12.06±2.59	0.998
Comorbidities:			
Acute exacerbation of chronic obstructive pulmonary disease alone	36	33	0.528
coexisting with cardiovascular disease	13	12	0.789
coexisting with metabolic syndrome	5	7	0.763
coexisting with anxiety/depression	6	5	0.763
coexisting with other conditions	4	6	0.744

**Table 3:** Clinical primary and secondary outcomes.

Primary and secondary outcome measure	Intervention group (*n* = 63)	Control group (*n* = 64)	Odds Ratio (95%CI)	*P* value
Average length of hospital stay (days)	9.79±5.84	12.67±5.87		0.006
Charges per case (¥)	8269.6±3609.84	10905.00±5342.12		0.001
Days of mechanical ventilation (days)	5.17±3.21 (*n* = 12)	8.64±4.58 (*n* = 14)		0.038
Ventilated/non-ventilated patients	12/51	14/50	0.84(0.354-1.994)	0.693
28-day mortality (all causes)	2/63 (1.22%)	0/64 (0%)	0.969(0.928-1.102)	0.496

**Table 4:** Clinical secondary outcomes.

Secondary outcome factors	Intervention group (*n* = 63)	Control group (*n* = 64)	*P* value
Heart rate (beats/min)			
Before intervention	95.56±17.31	94.69±12.64	0.748
72 h after intervention	76.57±7.08	82.19±14.53	0.007
Mean artery pressure (mmHg)			
Before intervention	93.87±13.45	91.28±12.50	0.263
72 h after intervention	88.83±7.46	88.02±8.55	0.57
Arterial partial pressure of oxygen (PaO_2_, mmHg)			
Before intervention	87.62±33.79	86.44±36.85	0.851
72 h after intervention	105.08±29.00	93.58±25.06	0.018
Arterial partial pressure of carbon dioxide (PaCO2, mmHg)			
Before intervention	45.67±12.30	48.20±18.03	0.358
72 h after intervention	43.20±7.62	45.93±11.10	0.109
Respiratory frequency (breaths/min)			
Before intervention	25.24±2.79	25.11±2.15	0.771
72 h after intervention	19.60±3.09	22.66±2.65	<0.001
Leucocyte count (10^9^/L)			
Before intervention	9.07±4.05	8.07±3.19	0.126
72 h after intervention	7.88±3.27	7.33±2.39	0.279
Neutrophil ratio (NEUT%)			
Before intervention	78.51±12.17	77.74±9.81	0.694
72 h after intervention	73.26±10.69	71.09±9.35	0.226
Platelet count (10^9^/L)			
Before intervention	142.25±44.03	133.97±42.68	0.284
72 h after intervention	140.89±47.99	117.61±33.91	0.002
Haemoglobin			
Before intervention	125.65±16.27	127.78±21.19	0.527
72 h after intervention	125.49±17.55	127.58±19.25	0.525
Creatinine (mmol/L)			
Before intervention	94.71±22.96	100.78±26.23	0.168
72 h after intervention	91.88±26.62	91.19±16.88	0.86
Alanine transaminase (ALT)			
Before intervention	25.17±12.00	26.23±12.32	0.622
72 h after intervention	27.17±11.77	28.63±14.59	0.539
Prothrombin time PT (s)			
Before intervention	12.54±1.17	12.50±1.20	0.861
72 h after intervention	12.67±1.01	12.65±1.08	0.88
Activated partial thromboplastin time APTT (s)			
Before intervention	30.42±3.82	29.74±4.06	0.33
72 h after intervention	30.15±3.16	30.20±2.77	0.932
Rate of voluntary termination of treatment (%)	4.76% (3/63)	9.38% (6/64)	0.492

## Data Availability

The data used to support the findings of this study are available from the corresponding author upon request.
